# Modulating efferocytosis in the intestinal epithelial cells during colorectal cancer

**DOI:** 10.3389/fonc.2026.1740918

**Published:** 2026-03-09

**Authors:** Laura Boeckaerts, Tania Løve Aaes, Evi Scheirlinckx, Sze Men Choi, Yunus Incik, Amanda Gonçalves, Tino Hochepied, Lars Vereecke, Kodi S. Ravichandran

**Affiliations:** 1 Vlaams Instituut voor Biotechnologie (VIB) Center for Inflammation Research, Ghent, Belgium; 2 Department of Biomedical Molecular Biology, Ghent University, Ghent, Belgium; 3 Vlaams Instituut voor Biotechnologie (VIB) Bioimaging Core, Ghent, Belgium; 4 Vlaams Instituut voor Biotechnologie (VIB) Transgenic Core, Ghent, Belgium; 5 Cancer Research Institute Ghent (CRIG), Ghent, Belgium; 6 Department of Internal Medicine and Pediatrics, Ghent University, Ghent, Belgium; 7 Division of Immunobiology, Department of Pathology and Immunology, Washington University School of Medicine, St. Louis, MO, United States

**Keywords:** Chimeric receptor, colorectal cancer, DSS colitis, efferocytosis, ELMO, SLC12A2, ZEB2

## Abstract

**Introduction:**

Colorectal cancer (CRC) is the thirdmost common cancer and a leading cause of cancer deaths worldwide, with over 1.9 million cases diagnosed in 2022. Due to poor response to classical cancer treatments, CRC is associated with low survival rates. This creates an urgent need for better understanding of CRC pathology. Cell death occurs continuously in solid tumors, and is also induced acutely, during chemotherapy. Dead cells are cleared by phagocytes via ‘efferocytosis’, an anti-inflammatory process that can lead to immune escape and reduced therapeutic efficacy. We hypothesized that efferocytosis might contribute to tumor development and that manipulating this process could be beneficial for CRC therapy.

**Materials and Methods:**

Here, we asked whether known approaches to enhance efferocytosis may alter disease parameters in experimental and genetic CRC mouse models. In the first approach, we chose transgenic expression of a chimeric efferocytosis receptor (BELMOTg) that removes dying cells in an anti-inflammatory manner, and in the second, we chose deleting a chloride transporter (Slc12a2KO) that increases efferocytosis but in a pro-inflammatory fashion.

**Results:**

Despite detectable expression of the transgenic proteins, many parameters of CRC including CRC pathogenesis were not significantly altered in mice with *BELMO* overexpression or *Slc12a2* knockout in the intestinal epithelial cells.

**Discussion:**

This suggests that these two approaches to clearing apoptotic cells is not sufficient to alter CRC progression. Targeting other phagocytic cell types or using other models of CRC might reveal a role (or otherwise) for efferocytosis in mitigating CRC in the future.

## Introduction

Intestinal epithelial cells (IEC), arising from stem cells at the base of the crypts, have a life cycle of just 4–5 days before they are replaced by newly generated epithelial cells (1). In humans, turnover in the healthy gut is high and estimated to be ~10 billion cells per day (2). The predominant mode of cell loss in the gut is thought to be passive cell shedding into the lumen. Still, a significant amount of dying cells is cleared through phagocytosis, either by macrophages and dendritic cells in the lamina propria (3) or by neighboring epithelial cells (4). Elimination of apoptotic cells is associated with active production of anti-inflammatory mediators and therefore contributes to the maintenance of immune tolerance as part of homeostasis in the gut (5).

Colorectal cancer (CRC) is the third most diagnosed cancer worldwide, after breast cancer and lung cancer, with almost 2 million new cases every year and the death rate representing around 9% of all cancer deaths (2022 data) (6). The current standard treatment for CRC is surgical resection, which may be complemented with neoadjuvant radio(chemo)therapy, depending on the stage of disease (7). While the use of immune checkpoint inhibitors, such as PD-1 blockade, has thus far been limited to the highly immunogenic microsatellite-instable (MSI) colorectal cancers (8), the large majority of cases (approximately 85%) are microsatellite-stable (MSS) tumors, which show little response to immunotherapies and have poor survival rates even after chemotherapy. Hence, a better understanding of the processes occurring within CRC is crucial.

The clearance of dying cells by phagocytes in the intestines could be important to regulate the immune tone within the tumor microenvironment. Yet, the complex network of interactions between the tumor, the microbiota, the intestinal epithelium, and the innate and adaptive immune system is yet to be fully defined.

We hypothesized that manipulating apoptotic cell uptake by epithelial cells in the colonic tissue may influence the development of CRC. This hypothesis was based on two recent findings from our laboratory. We recently generated a novel chimeric phagocytic receptor, denoted BELMO, which is a fusion between the phosphatidylserine receptor BAI1 (important for apoptotic cell recognition) and the cytoplasmic adaptor protein ELMO1 (9). First, overexpression of the BELMO receptor in phagocytes results in enhanced efferocytosis and could mitigate inflammatory insults during DSS-induced colitis as well as acute kidney injury. Second, transcriptomic analysis (RNA-Seq) of efferocytic phagocytes revealed pronounced changes in expression of many solute carrier (SLC) genes (“SLC program”) induced by apoptotic cell engulfment (10). Modulating the expression of some of these SLCs resulted in altered efferocytosis exemplified by knock-out of the chloride transporter Slc12a2, which led to increased apoptotic cell engulfment in macrophages and a fibroblast cell line; this implied that Slc12a2 acts as a brake on efferocytosis (11). Importantly, in contrast to the anti-inflammatory response seen with BELMO-expressing efferocytic phagocytes, deleting Slc12a2 expression induced increased efferocytosis that accompanied a pro-inflammatory response. During the course of the studies with Slc12a2, we also noted that another related channel, Slc12a4, acts as a promoter of efferocytosis, and its loss led to a decrease in efferocytosis. Here, we evaluate these complementary approaches in the context of disease onset and progression in CRC.

## Results

### BELMO expression in intestinal epithelial cells in the AOM/DSS model

**Figure 1:**
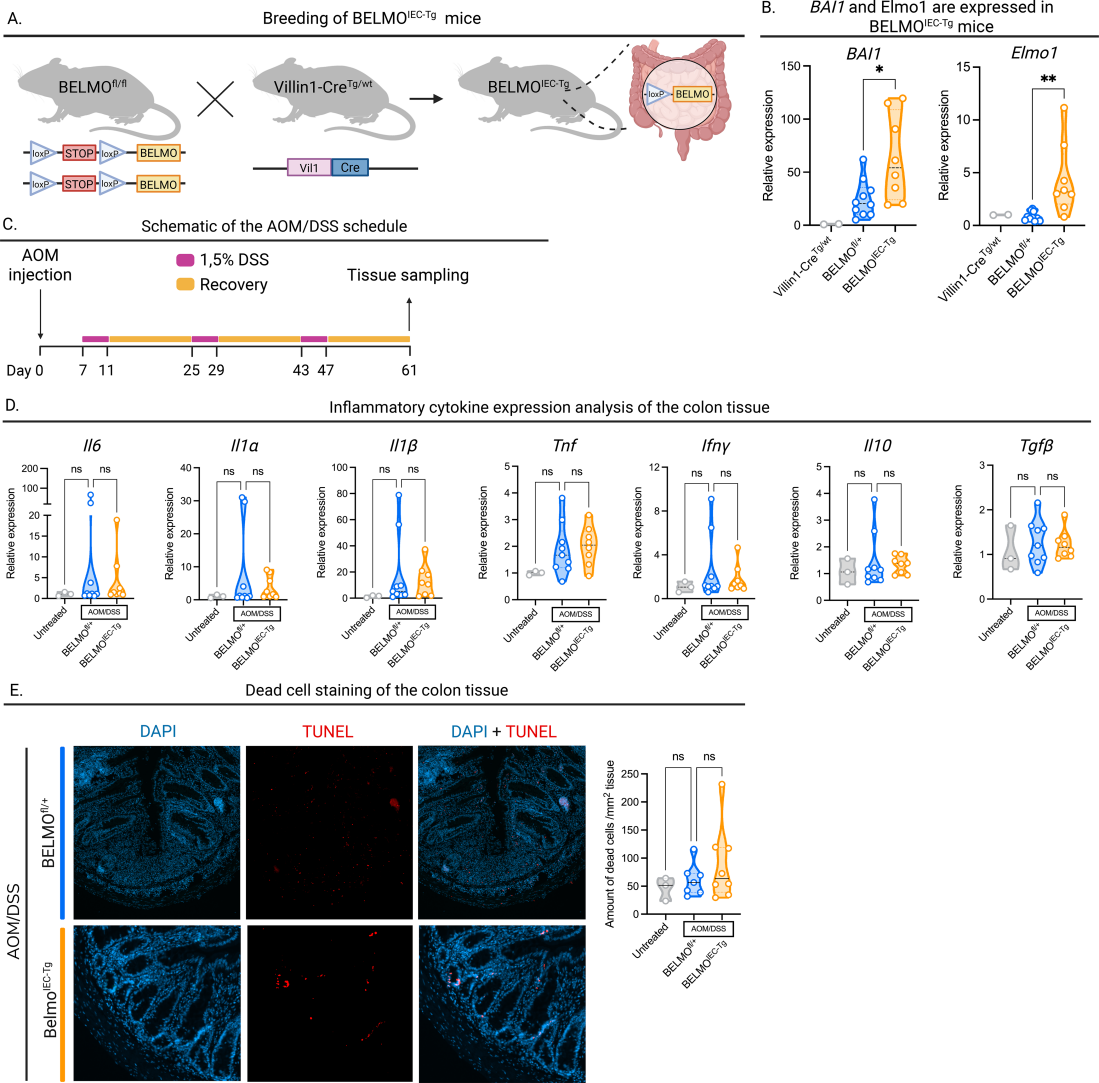
Expression of BELMO in the intestinal epithelial cells. **(A)** Graphical representation of the mousecrosses to achieve *BELMO^IEC-Tg^* mice. Made with BioRender.com. **(B)** BAI1 and Elmo1 expression in the colon tissue of *BELMO^IEC-Tg^* mice confirmed by RT-qPCR. **(C)** Schematic overview of the AOM/DSS treatment used in this study. Made with BioRender.com. **(D)** Inflammatory cytokine measurements by RT-qPCR. Colon tissue from BELMOIEC-Tg mice (n=8) treated with AOM/DSS and Cre-negative mice (n=9) treated with AOM/DSS or untreated mice (n=3) was lysed, RNA was isolated and analyzed by RT-qPCR. **(E)** Left: representative DAPI and TUNEL stained images of colon sections from untreated (n=3), and AOM/DSS-treated *BELMO^IEC-Tg^* (n=8) and Cre-negative mice (n=3) at the end of treatment. Right: TUNEL positive cells per colon tissue section area (mm2). Each symbol represents one mouse. Data are represented as mean ± SEM. Statistical significance was determined by one-way ANOVA **(B, D, E)**. Ns, non-significant; *p ≤ 0.05; **p ≤ 0.01.

To address whether BELMO expression in intestinal epithelial cells would modify disease parameters in the azoxymethane/dextran sodium sulfate (AOM/DSS) model, we targeted *BELMO* transgenic expression to murine intestinal epithelial cells by crossing *Villin1-Cre* mice with *BELMO^fl-STOP-fl^* transgenic mice (*BELMO^IEC-Tg^* mice, **Figure 1A**), thereby aiming to address anti-inflammatory enhancement of efferocytosis in CRC. The resulting *BELMO^IEC-Tg^* mice were viable and showed no overt phenotype under basal conditions. We confirmed increased expression of *BAI1* and *Elmo1*, the two components of *BELMO*, in colon lysates (**Figure 1B**).

Transgenic BELMO expression is known to be anti-inflammatory and attenuate multiple inflammatory insults in mice, including acute kidney injury, DEN-induced liver toxicity and DSS-induced colitis (9). However, in absence of injury or disease, *BELMO^IEC-Tg^* mice showed no changes in basal cytokine expression. In the purpose of this work, we chose a well-established colitis-induced CRC model. The AOM/DSS model is a chemically-induced model of CRC, which relies on DNA damage caused by a single injection of AOM, followed by repeated cycles of colitis induced by DSS (12) (**Figure 1C**).

Initially, we investigated whether BELMO expression changed the inflammatory environment induced by the AOM/DSS treatment. For this purpose, we isolated colons of *BELMO^IEC-Tg^* mice and BELMO^fl/+^ littermate control mice (hereafter referred to as Cre-negative), that were treated with AOM/DSS, at day 61 after AOM/DSS initiation. *Villin1-Cre* littermate control mice were left untreated and sacrificed on the same day (hereafter referred to as untreated). We chose this timepoint to assess the effect of tumor growth and chronic inflammation associated with cancer, rather than the acute inflammation induced by DSS alone.

We divided the distal half of the colon into three segments: One part was used for microscopy, another for cytokine production analysis by ELISA, and the remaining part was lysed for cytokine expression analysis by RT-qPCR. We investigated the expression of both pro-inflammatory cytokines such as *Il6*, *Il1*α, *Il1*β, *Inf*γ and *Tnf*, as well as anti-inflammatory cytokines *Il10* and *Tgf*β, which are known to be involved in either CRC, efferocytosis, or both. At the transcript level, we did not observe any statistically significant differences in the expression of any of these cytokines between *BELMO^IEC-Tg^* and Cre-negative mice (**Figure 1D**). These results were confirmed by ELISA using the conditioned medium from 18h colon explant cultures (**Supplementary Figure S2**).

Next, we determined whether intestinal epithelial cells expressing *BELMO* could engulf more apoptotic cells compared to Cre-negative control epithelia. TUNEL staining, which specifically labels apoptotic cells, can serve as an indirect readout of efferocytosis on tissue sections. A higher number of apoptotic cells in the tissue may indicate either increased induction of cell death, or *a* reduced clearance efficiency. Using this method, we visualized non-engulfed apoptotic cells in the colonic tissue of *BELMO^IEC-Tg^* and Cre-negative mice following AOM/DSS treatment at day 61. However, surprisingly, we did not observe any differences in the number of apoptotic cells (**Figure 1E**). Of note, we also did not detect a significant increase in apoptotic cells in AOM/DSS-treated versus untreated mice.

**Figure 2:**
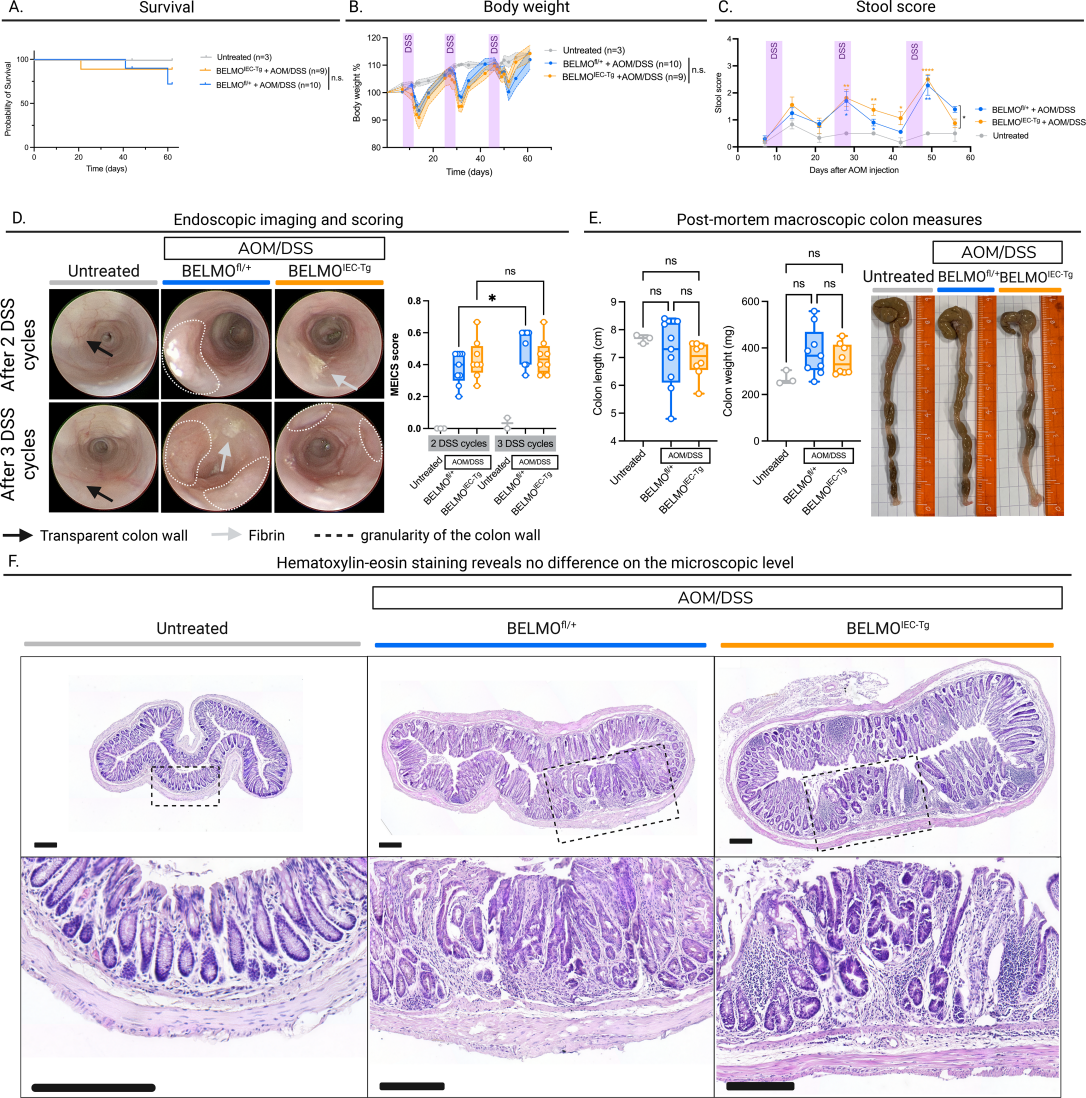
Expression of *BELMO* to enhance efferocytosis does not significantly change disease progression in an AOM/DSS model of CRC. **(A)** Kaplan-Meier survival curves of untreated, and AOM/DSStreated *BELMO^IEC-Tg^* and Cre-negative mice. **(B)** Changes in body weights of untreated, and AOM/DSStreated *BELMO^IEC-Tg^* and Cre-negative mice during the follow-up period. **(C)** Clinical stool scores over the follow-up period of untreated, and AOM/DSS-treated *BELMO^IEC-Tg^* and Cre-negative mice based on the hemocare fecal test. **(D)** representative images of colonoscopy performed in untreated, and AOM/DSStreated *BELMO^IEC-Tg^* and Cre-negative mice after recovery of the second (between day 40-42, upper row) and third (between day 55-60, bottom row) DSS cycle (left). Progression of the MEICS score from DSS cycle 2 to 3 quantified (right). **(E)** Colon length (left), colon weight (middle) and representative macroscopic images (right) of untreated,and AOM/DSS-treated *BELMO^IEC-Tg^* and Cre-negative mice at the end of the AOM/DSS treatment (=day 61). **(F)** representative Hematoxylin-eosin staining on colon sections of untreated (left), and AOM/DSS-treated *BELMO^IEC-Tg^* (right) and Cre-negative mice (middle) at the end of the treatment. Bottom: magnified images of the area defined by the dashed black boxes. Scale bar = 200 um. **(A–C)** untreated: n=3, AOM/DSS-treated *BELMO^IEC-Tg^*: n=9 and Cre-negative mice: n=10 **(D, E)** untreated: n=3, AOM/DSS-treated *BELMO^IEC-Tg^*: n=8 and Cre-negative mice: n=9. Each symbol represents one mouse. Data are represented as mean ± SEM. Statistical significance was determined by Log-rank Mantel-Cox test **(A)**, REML variance components analysis **(B)**, Tukey’s multiple comparisons test **(C)** or one-way ANOVA **(D, E)**. Ns, non-significant; *p ≤ 0.05.

Mice treated with AOM/DSS typically develop significant tumors after the second DSS cycle and the disease progressively worsens after the third DSS cycle (12). We evaluated the pathophysiology of *BELMO^IEC-Tg^* mice *in vivo* by monitoring their overall survival (**Figure 2A**), body weight (**Figure 2B**), stool score (**Figure 2C**) and intestinal tissue health and tumor development (**Figure 2D**). Based on the survival curves and body weight changes, we did not observe any overall disease differences between *BELMO^IEC-Tg^* and Cre-negative littermate control mice treated with AOM/DSS (**Figures 2A, B**). We further evaluated the intestinal pathology, first based on the texture and blood content of stool. While the stool score of mice treated with AOM/DSS was significantly higher than that of untreated mice, *BELMO^IEC-Tg^* mice displayed an improvement in intestinal health over wild-type mice only at the final time point of measurement (**Figure 2C**). Secondly, we performed high-resolution endoscopy for *in vivo* intestinal pathology scoring after the second and third DSS cycles. To assess disease severity, we applied the murine endoscopic index of colitis severity (MEICS) scoring method (13) and evaluated disease progression from the second to the third DSS cycle. Our endoscopic images revealed a trend toward slower CRC progression in *BELMO^IEC-Tg^* compared to Cre-negative littermates following AOM/DSS treatment (**Figure 2D**), with a milder increase in epithelial thickness, granularity, fibrin & blood spots from 2 to 3 rounds of DSS treatment. Finally, macroscopic measurements of the colon length and weight were performed *post-mortem*, at day 61, which revealed no significant differences between *BELMO^IEC-Tg^* and Cre-negative mice (**Figure 2E**). These observations were further supported by a hematoxylin-eosin staining of colon sections of untreated mice and AOM/DSS-treated Cre-negative and *BELMO^IEC-Tg^* mice. This histopathological analysis showed thickening of the colon wall in both Cre-negative and *BELMO^IEC-Tg^* mice (**Figure 2F**). Furthermore, both genotypes show severe epithelial dysplasia with a loss of normal crypt structure, whereas untreated mice show regular crypt structure.

Collectively, these data suggest that targeted BELMO expression in the intestinal epithelial cells did not influence disease progression in the inflammation-associated AOM/DSS model of CRC in *BELMO^IEC-Tg^* mice compared to Cre-negative littermates.

### *Slc12a2^IEC-cKO^* increases efferocytosis *in vivo*, but does not influence CRC pathophysiology

**Figure 3:**
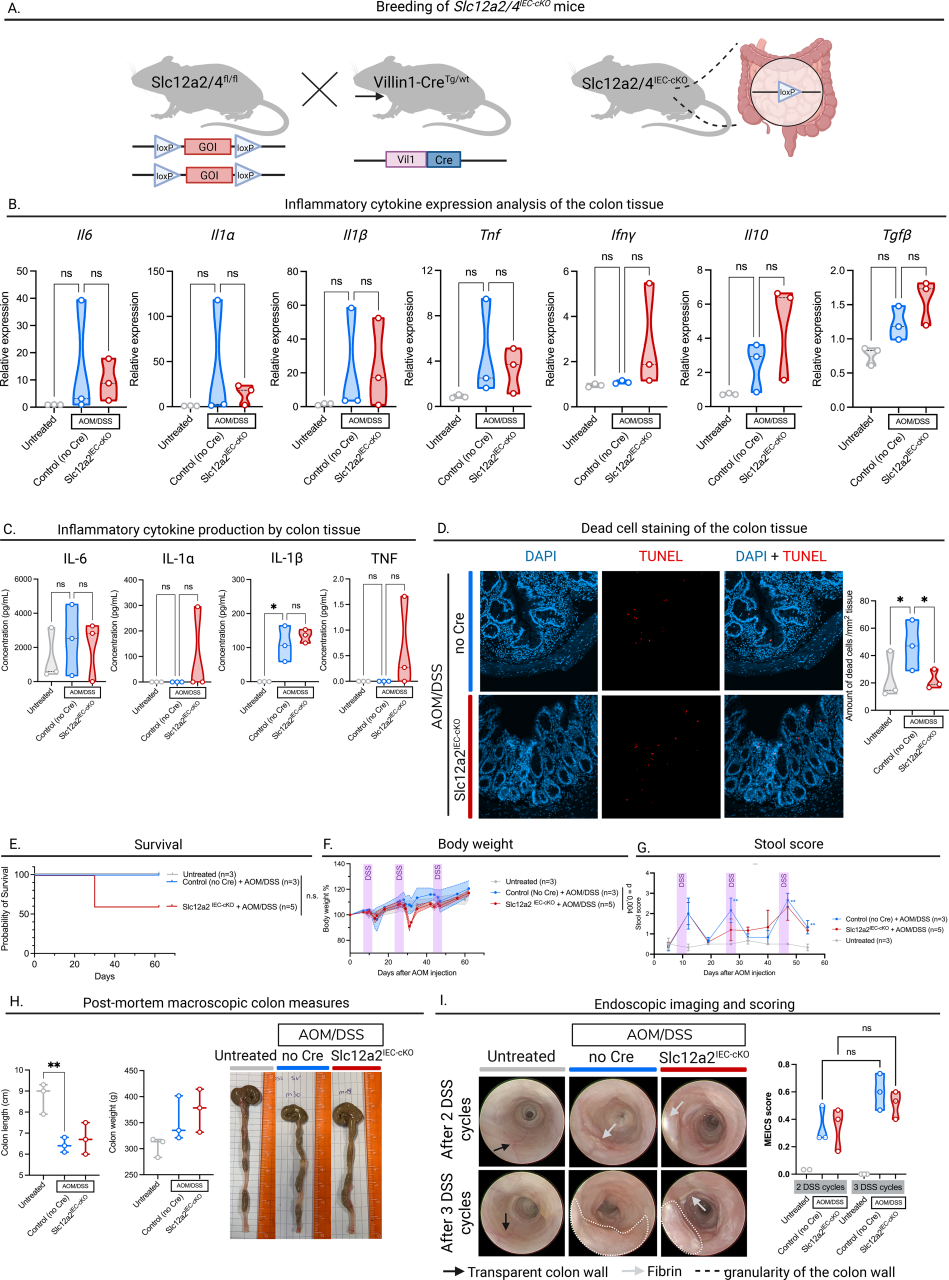
Slc12a2^IEC-cKO^ increases efferocytosis in vivo but does not influence CRC pathophysiology. **(A)** Graphical representation of the mouse crosses to achieve Slc12a2^IEC-cKO^ and *Slc12a4^IEC-cKO^* mice. Made with BioRender.com. **(B)** Inflammatory cytokine measurements by RT-qPCR. Colon tissue from Slc12a2^IEC-cKO^ mice treated with AOM/DSS and Cre-negative mice treated with AOM/DSS or untreated mice was lysed, RNA was isolated and analyzed by RT-qPCR. **(C)** Inflammatory cytokine measurements by ELISA. Colon tissue from Slc12a2^IEC-cKO^ mice treated with AOM/DSS and Cre-negative mice treated with AOM/DSS or untreated mice were isolated and put in culture overnight. Inflammatory cytokines were measured in the supernatant. **(D)** Left: representative DAPI and TUNEL stained images of colon sections from untreated, and AOM/DSS-treated Slc12a2^IEC-cKO^ and Cre-negative mice at the end of treatment. Right: TUNEL positive cells per colon tissue section area (mm2). **(E)** Kaplan-Meier survival curves of untreated, and AOM/DSS-treated Slc12a2^IEC-cKO^ and Cre-negative mice. **(F)** Changes in body weights of untreated, and AOM/DSS-treated Slc12a2^IEC-cKO^ and Cre-negative mice during the follow-up period. **(G)** Clinical stool scores over the follow-up period of untreated, and AOM/DSS-treated Slc12a2^IEC-cKO^ and Cre-negative mice based on the hemocare fecal test. **(H)** Colon length (left), colon weight (middle) and representative macroscopic images of untreated,and AOM/DSS-treated Slc12a2^IEC-cKO^ and Cre-negative mice at the end of the AOM/DSS treatment (=day 61). **(I)** Representative images of colonoscopy performed in untreated, and AOM/DSS-treated Slc12a2^IEC-cKO^ and Cre-negative mice after recovery of the second (between day 40-42, upper row) and third (between day 56-58, bottom row) DSS cycle (left). Progression of the MEICS score from DSS cycle 2 to 3 quantified (right). **(B–D, H, I)** untreated: n=3, AOM/DSS-treated Slc12a2^IEC-cKO^: n=3 and Cre-negative mice: n=3 **(E–G)** untreated: n=3, AOM/DSS-treated Slc12a2^IEC-cKO^: n=5 and Cre-negative mice: n=3. Each symbol represents one mouse. Data are represented as mean ± SEM. Statistical significance was determined by one-way ANOVA **(B–D, H, I)**, by Log-rank Mantel-Cox test **(E)**, REML variance components analysis **(F)**, or Tukey’s multiple comparisons test **(G)**. Ns, non-significant; *p ≤ 0.05; **p ≤ 0.01.

It has previously been shown that loss of Slc12a2 transporter increases efferocytosis, but this is associated with a pro-inflammatory signature (11). In the context of intestinal epithelial cells, Slc12a2 is expressed on the basolateral membrane, where it participates in fluid secretion (14, 15). Therefore, in a second model of CRC, to study pro-inflammatory efferocytosis in the colon, we generated mice with a targeted deletion of Slc12a2 in intestinal epithelial cells (*Slc12a2^IEC-cKO^*) by crossing *Slc12a2^fl/fl^* mice with *Villin1-Cre* (*Vil1-Cre*) mice (**Figure 3A**). The resulting mice were viable and displayed no overt abnormalities. Genotyping data from tail biopsies and RT-qPCR validation on bulk colon tissue confirming the knock-out in the intestines are shown in **Supplementary Figures S2A, B** (**Supplementary Figures S4A, B**).

To investigate the role of pro-inflammatory efferocytosis in colitis-associated CRC, we subjected the *Slc12a2^IEC-cKO^* to the AOM/DSS model of colorectal cancer. Since reduction of Slc12a2 expression has been reported to induce inflammation, we first assessed whether knock-out of *Slc12a2* in intestinal epithelial cells would exacerbate the inflammatory environment induced by the AOM/DSS model. For this purpose, we isolated colons from *Slc12a2^IEC-cKO^* and Cre-negative control mice, that were both treated with AOM/DSS. Analogous to the approach used for BELMO^IEC-Tg^ mice, we divided the distal half of the colon in three sections, each used for microscopy, cytokine analysis by ELISA, or cytokine expression analysis by RT-qPCR (**Figure 1C**). While the expression of inflammatory cytokines seems to remain unchanged between *Slc12a2^IEC-cKO^* and Cre-negative control mice in our setup (**Figure 3B**), cytokine levels measured in the supernatant of colonic explants (18h) also appear to show no significant differences between the inflammatory signatures of our modest cohort of *Slc12a2^IEC-cKO^* and Cre-negative control mice. (**Figure 3C**).

To assess the number of non-engulfed apoptotic cells (which often turn necrotic and are detected via the TUNEL assay) in colonic tissue at day 61 after AOM/DSS initiation, we performed TUNEL staining (**Figure 3D**). This suggests a significant increase in dead cell numbers in Cre-negative control mice treated with AOM/DSS compared to their untreated littermates, while this increase in dying cells was significantly reduced in the *Slc12a2^IEC-cKO^* mice. Thus, loss of *Slc12a2* in intestinal epithelial cells appear to enhance apoptotic cell uptake in the colon.

When the *Slc12a2^IEC-cKO^* and Cre-negative control mice were treated with AOM/DSS, they started to develop colonic tumors after the second DSS cycle. To assess the pathophysiology of these mice *in vivo*, we monitored survival (**Figure 3E**), body weight (**Figure 3F**) and stool score (**Figure 3G**). Although we observed an overall lower survival rate in *Slc12a2^IEC-cKO^* mice compared to Cre-negative control mice treated with AOM/DSS, the difference did not reach statistical significance. This trend may be partially explained by a more pronounced drop in body weight after the second DSS cycle, which was more evident in *Slc12a2^IEC-cKO^* mice. However, when examining intestinal pathology using the stool scoring method, we did not observe any differences between AOM/DSS-treated Cre-negative control and *Slc12a2^IEC-cKO^* mice. Macroscopic measurements of the intestines, including colon length and colon weight measured at day 61 post-AOM/DSS initiation, also showed no differences between Cre-negative control and Slc12a2^IEC-cKO^ mice in our setup (**Figure 3H**). We also performed endoscopic analysis after the second and third DSS cycles. Both Cre-negative control and *Slc12a2^IEC-cKO^* mice treated with AOM/DSS showed signs of intestinal pathology already after the second DSS cycle, which further increased after the third cycle. However, until now, we did not observe any significant differences in disease progression between Cre-negative control and *Slc12a2^IEC-cKO^* mice (**Figure 3I**). Finally, microscopic imaging showed significant dysplasia, characterized by colon wall thickening and aberrant crypt architecture, in both Cre-negative control and *Slc12a2^IEC-cKO^* mice treated with AOM/DSS, compared to untreated mice (**Supplementary Figure S4C**). Collectively, we observed only minor differences in the aggravation of general disease parameters in *Slc12a2^IEC-cKO^* mice compared to Cre-negative control mice, and we do not see notable differences in intestinal disease parameters in the conditions tested.

### *Slc12a4^IEC-cKO^* decreases efferocytosis *in vivo*, but does not influence CRC pathophysiology

**Figure 4:**
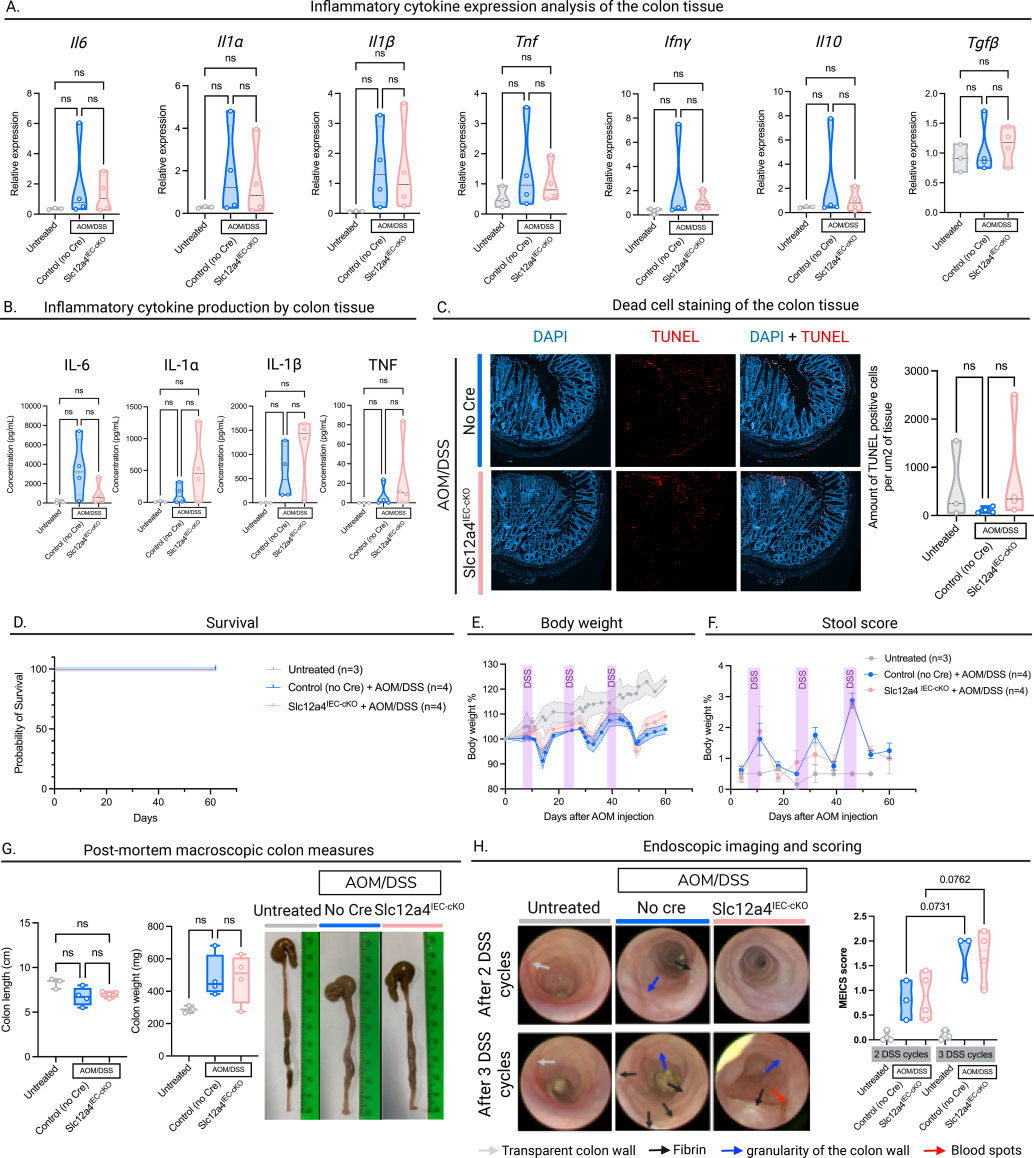
Slc12a4^IEC-cKO^ increases efferocytosis in vivo but does not influence CRC pathophysiology. **(A)** Inflammatory cytokine measurements by RT-qPCR. Colon tissue from Slc12a4^IEC-cKO^ mice treated with AOM/DSS and Cre-negative mice treated with AOM/DSS or untreated mice was lysed, RNA was isolated and analyzed by RT-qPCR. **(B)** Inflammatory cytokine measurements by ELISA. Colon tissue from Slc12a4^IEC-cKO^ mice treated with AOM/DSS and Cre-negative mice treated with AOM/DSS or untreated mice were isolated and put in culture overnight. Inflammatory cytokines were measured in the supernatant. **(C)** Left: representative DAPI and TUNEL stained images of colon sections from untreated,and AOM/DSS-treated Slc12a4^IEC-cKO^ and Cre-negative mice at the end of treatment. Right: TUNEL positive cells per colon tissue section area (mm^2^). **(D)** Kaplan-Meier survival curves of untreated, and AOM/DSS-treated Slc12a4^IEC-cKO^ and Cre-negative mice. **(E)** Changes in body weights of untreated, and AOM/DSS-treated Slc12a4^IEC-cKO^ and Cre-negative mice during the follow-up period. **(F)** Clinical stool scores over the follow-up period of untreated, and AOM/DSS-treated Slc12a4^IEC-cKO^ and Cre-negative mice based on the hemocare fecal test. **(G)** Colon length (left), colon weight (middle) and representative macroscopic images of untreated, and AOM/DSS-treated Slc12a4^IEC-cKO^ and Cre-negative mice at the end of the AOM/DSS treatment (=day 61). **(H)** Representative images of colonoscopy performed in untreated, and AOM/DSS-treated Slc12a4^IEC-cKO^ and Cre-negative mice after recovery of the second (between day 40-42, upper row) and third (between day 56-58, bottom row) DSS cycle (left). Progression of the MEICS score from DSS cycle 2 to 3 quantified (right). Untreated: n=3, AOM/DSS-treated Slc12a4^IEC-cKO^: n=4 and Cre-negative mice: n=4. Each symbol represents one mouse. Data are represented as mean ± SEM. Statistical significance was determined by one-way ANOVA **(A–C, G, H)**, Log-rank Mantel-Cox test **(D)**, REML variance components analysis **(E)**, or Tukey’s multiple comparisons test **(F)**. Ns, non-significant.

Since boosting efferocytosis did not have a significant effect on CRC pathophysiology, we next evaluated whether the opposite phenotype, impaired efferocytosis, might influence the disease. Therefore, we generated a mouse model with decreased efferocytic capacity by knocking out *Slc12a4*, which is a positive regulator of efferocytosis (11). We created Slc12a4 knock-out mice specifically in intestinal epithelial cells (*Slc12a4^IEC-cKO^)* by crossing *Slc12a4^fl/fl^* mice with *Vil1-Cre* mice (**Figure 3A**). Although only about half of the mice survived to early adulthood, the survivors were healthy and did not display overt pathology. Genotyping results from tail biopsies are shown in **Supplementary Figure S3A** (**Supplementary Figure S5A**). The effect of *Slc12a4* deficiency on the inflammatory response after engulfment is not known. Therefore, we investigated whether knock-out of *Slc12a4* in intestinal epithelial cells would change the inflammatory environment induced by AOM/DSS. We isolated colons from Slc12a4^IEC-cKO^ and Cre-negative control mice that were challenged with AOM/DSS treatment. Again, the colon was divided into three readouts, one of which was cytokine expression analysis. The expression of key pro- and anti-inflammatory cytokines remained unchanged in colon lysates (**Figure 4A**). Analysis of cytokine levels in the supernatant of colonic explants further confirmed no significant differences on the protein level (**Figure 4B**).

Next, we assessed the number of non-engulfed apoptotic cells in the colonic tissue by TUNEL staining at day 61 after AOM/DSS initiation (**Figure 4C**). This indirect measure of efferocytosis revealed a trend towards increased apoptotic cell numbers in Slc12a4^IEC-cKO^ mice compared to Cre-negative mice, although it did not appear statistically significant. This finding is consistent with our previous report showing that knock-out of Slc12a4 decreases apoptotic cell uptake *in vitro*.

To investigate whether Slc12a4 is involved in CRC initiation and progression, *Slc12a4^IEC-cKO^* and Cre-negative control mice were treated with AOM/DSS as shown in **Figure 1C**. The pathophysiology of the mice was closely monitored *in vivo* by assessing survival, body weight and stool score. All the mice that underwent AOM/DSS treatment survived until the experimental endpoint (**Figure 4D**). We did not observe any differences in body weight trends between *Slc12a4^IEC-cKO^* and Cre-negative control mice during the procedure (**Figure 4E**). Looking more specifically at intestinal pathology, we evaluated the fecal content of the mice for consistency and presence of blood. We observed a slightly blunted peak in stool score after the second DSS cycle in *Slc12a4^IEC-cKO^* mice compared to Cre-negative controls, however, this trend was not maintained during the third DSS cycle (**Figure 4F**). Next, we examined macroscopic parameters of the intestines after dissection at day 61. While we observed colon shortening in mice treated with AOM/DSS compared to untreated mice, there were no differences in colon length between *Slc12a4^IEC-cKO^* and Cre-negative control mice treated with AOM/DSS (**Figure 4G**). Colon weight showed the opposite trend, with a modest increase (p=0,06) in the treated groups compared to untreated, but no difference between the two treated genotypes. We again performed endoscopy after the second and third cycle of DSS and scored pathology using the MEICS scoring system. Both AOM/DSS-treated Cre-negative control and *Slc12a4^IEC-cKO^* mice showed similar progression of pathology from DSS cycle 2 to DSS cycle 3, while the scores of untreated mice remain unchanged (**Figure 4H**). At the microscopic level, histopathology of the colon revealed significant dysplasia, thickening of the colon wall, and loss of crypt structure in both Cre-negative control and *Slc12a4^IEC-cKO^* mice treated with AOM/DSS, while we see normal thickness and regular crypt structure in untreated mice (**Supplementary Figure S5B**). However, no differences in the extent of dysregulation were observed in *Slc12a4^IEC-cKO^* and control mice.

Thus, when we knock-out *slc12a4* in the intestinal epithelial cells, the number of residual dead cells in the colon tissue tends to marginally increase, which suggests cautiously a decreased efferocytic capacity in *slc12a4*-deficient cells. However, we do not see differences in colon cancer progression in Slc12a4^IEC-cKO^ mice compared to Cre-negative control mice.

### Boosting anti-inflammatory efferocytosis does not influence CRC pathophysiology in a genetic CRC model

Since CRC comprises multiple subtypes, we aimed to extend our validation beyond the inducible colitis-associated cancer model. Therefore, we transgenically expressed *Zeb2* in the intestinal epithelial cells of *Belmo^IEC-Tg^* mice. Expression of the epithelial-to-mesenchymal transition (EMT)-regulator Zeb2 in intestinal epithelial cells, spontaneously induces intestinal barrier disruption, chronic inflammation and microbiota dysbiosis (16). As early as five weeks of age, these mice develop diarrhea, and around the age of 18 weeks, they exhibit significant body weight loss due to invasive and microbiota-driven CRC development in the proximal colon.

**Figure 5:**
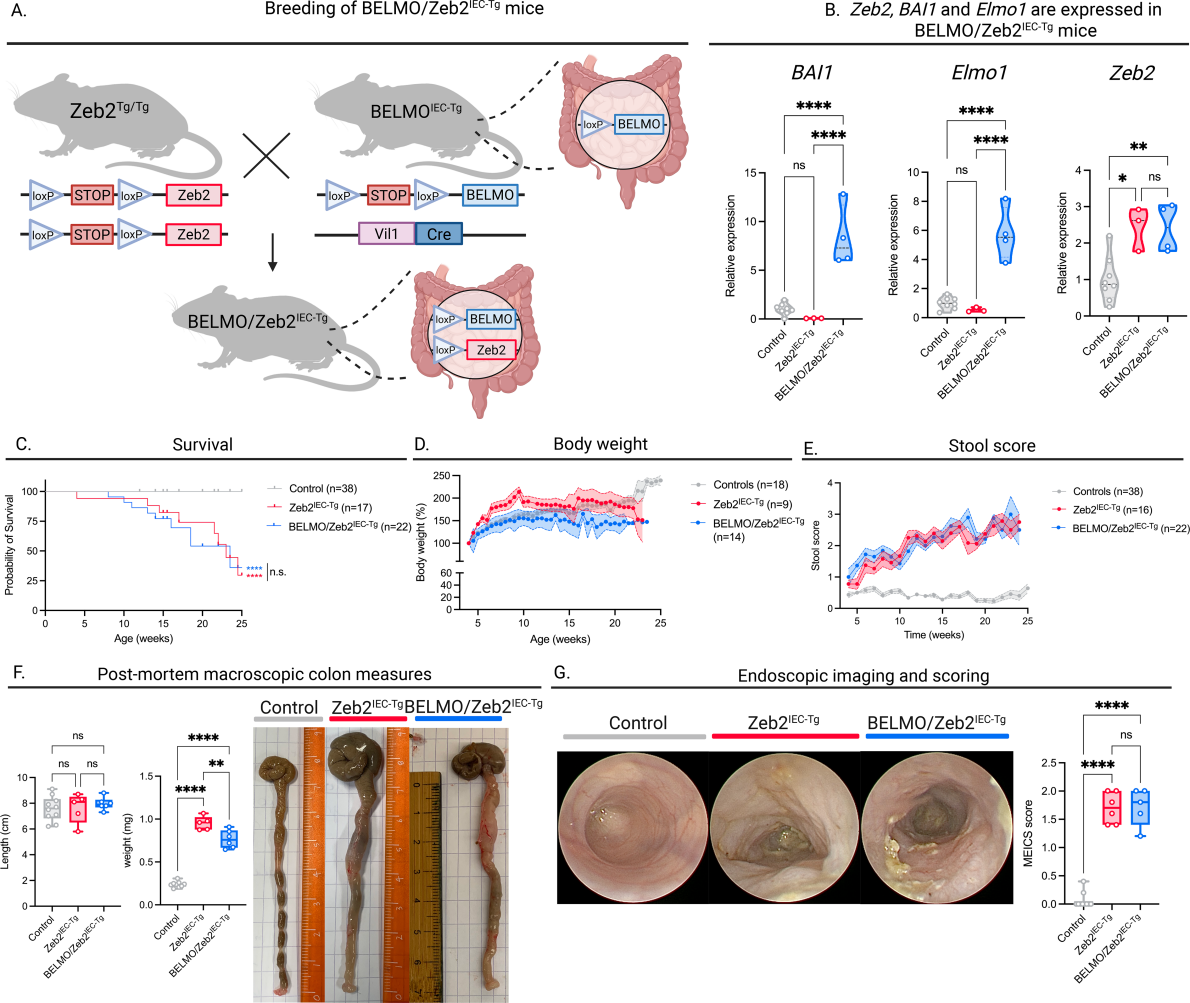
Boosting anti-inflammatory efferocytosis in a spontaneous model of CRC. **(A)** Graphical representation of the mouse crosses to achieve BELMO/Zeb2^IEC-Tg^ mice. Made with BioRender.com. **(B)** Zeb2, BAI1 and Elmo1 expression in the colon tissue of BELMO/Zeb2^IEC-Tg^ mice confirmed by RT-qPCR. **(C)** Kaplan-Meier survival curves of control (n=38), Zeb2^IEC-Tg^ (n=17) and BELMO/Zeb2^IEC-Tg^ (n=22) mice. Stars indicate significance between the grey line and the respective color. **(D)** Changes in body weights of control (n= 18), Zeb2^IEC-Tg^ (n=9) and BELMO/Zeb2^IEC-Tg^ (n=14) mice during the follow-up period (25 weeks). **(E)** Clinical stool scores over the follow-up period of control (n=38), Zeb2^IEC-Tg^ (n=17) and BELMO/Zeb2^IEC-Tg^ (n=22) mice based on the hemocare fecal test. **(F)** Colon length (left), colon weight (middle) and representative macroscopic images (right) of control (n=10), Zeb2^IEC-Tg^ (n=5) and BELMO/Zeb2^IEC-Tg^ (n=6) mice at 15 weeks of age. **(G)** Representative images of colonoscopy performed in control (n=7), Zeb2^IEC-Tg^ (n= 6) and BELMO/Zeb2^IEC-Tg^ (n=5) mice at 15 weeks of age. Quantification of the MEICS score at 15 weeks (right). Each symbol represents one mouse. Data are represented as mean ± SEM. Statistical significance was determined by one-way ANOVA **(B, F, G)**, Log-rank Mantel-Cox test **(C)**, REML variance components analysis **(D)**, or Tukey’s multiple comparisons test **(E)**. Ns, non-significant; *p ≤ 0.05; **p ≤ 0.01; ***p ≤ 0.001; ****p ≤ 0.0001.

We generated a spontaneous CRC model with increased efferocytosic capacity in intestinal epithelial cells by crossing conditional *Zeb2^Tg/Tg^* with *BELMO^IEC-Tg^* mice (**Figure 5A**). The resulting offspring consisted of *Zeb2^IEC-Tg^*, *BELMO/Zeb2^IEC-Tg^* and wild-type littermate controls. We first validated transgenic expression of *BELMO* and *Zeb2* in intestinal tissue by performing RT-qPCR for *BAI1*, *Elmo1* and *Zeb2* on tissue lysates. We confirmed that *BAI1* and *Elmo1* were only expressed in *BELMO/Zeb2^IEC-Tg^* mice and that *Zeb2* was highly expressed in *Zeb2^IEC-Tg^* and *BELMO/Zeb2^IEC-Tg^* mice compared to control mice were *Zeb2* expression was low (**Figure 5B**). However, *Zeb2* RNA expression can be present in the colon of healthy mice at variable levels, exclusively localized to mesenchymal cells (Human Protein Atlas), which we also observe in our control mice. In the context of intestinal inflammation and CRC, *Zeb2* expression can be found in epithelial cells, thereby promoting EMT.

Assessing general disease parameters, we did not observe a difference in survival (**Figure 5C**) or body weight (**Figure 5D**) between *Zeb2^IEC-Tg^* and *BELMO/Zeb2^IEC-Tg^* mice. We also assessed additional parameters more specific for intestinal disease. We determined fecal consistency and presence of blood, combined as the stool score. We did not detect a difference in stool scores between *Zeb2^IEC-Tg^* and *BELMO/Zeb2^IEC-Tg^* mice up until 25 weeks of age.

Next, we studied ex vivo measures of the colon at 15 weeks. Although the *Zeb2^IEC-Tg^* model of CRC is dependent on intestinal inflammation, we did not observe differences in colon length between Zeb2^IEC-Tg^ and control mice, nor between Zeb2^IEC-Tg^ and *BELMO/Zeb2^IEC-Tg^* mice. The colon weight, however, only differed significantly, with an increase in both *Zeb2^IEC-Tg^* and *BELMO/Zeb2^IEC-Tg^* mice compared to healthy controls. Moreover, colons of *BELMO/Zeb2^IEC-Tg^* were significantly lighter than those *of Zeb2^IEC-Tg^* mice. To investigate this in more detail, we performed endoscopy at 15 weeks of age and quantified intestinal disease by the MEICS scoring system. No difference in MEICS scores of were observed between *Zeb2^IEC-Tg^* and *BELMO/Zeb2^IEC-Tg^* mice, but both groups scored significantly higher than healthy control mice. Thus, we conclude that there are no major differences in CRC progression between *Zeb2^IEC-Tg^* and *BELMO/Zeb2^IEC-Tg^* mice.

## Discussion

Our original hypothesis for this study was that *BELMO* expression would induce anti-inflammatory efferocytosis (9), thereby creating an immunosuppressive environment and promoting a faster-growing tumor phenotype, whereas the absence of *Slc12a2* would induce pro-inflammatory efferocytosis (11) that might slow tumor progression. We surmised that our experimental design, which was developed to capture both phenotypes, would provide novel insights into CRC research. Previous studies have mostly suggested that increased efferocytosis or increased expression of efferocytosis-related molecules promote tumor progression. For example, Sun Mi Hong and colleagues suggest that Nicotinamide phosphoribosyltransferase (NAMPT), a metabolic enzyme and key regulator of inflammation, drives efferocytic activity and M2-like polarization of tumor-associated macrophages, thereby potentiating CRC progression (17). Moreover, Zhixin Ma et al. proposed that extracellular vesicles, derived from apoptotic CRC cells contain MFGE8, which may promote uptake of apoptotic cells by neighboring macrophages through the αvβ3-STAT3 signaling pathway (18). They further reason that the clearance of apoptotic cells, after cisplatin treatment, contributes to cisplatin resistance.

A number of diseases are associated with impaired efferocytosis capacity and can evoke unusually high numbers of dead cells (19). Among these diseases are chronic obstructive lung disease (COPD), where alveolar macrophages show reduced efferocytosis capacity, or cardiovascular diseases such as atherosclerosis but also cancer. In our experiments, we did not observe any effect of altered epithelial efferocytosis on the development and/or progression of CRC. We specifically chose to manipulate efferocytosis capacity in intestinal epithelial cells, as these cells are known to clear dying neighboring cells and are relevant in radiation- and DSS-induced colitis. Although their clearance capacity is lower than that of professional phagocytes, their close proximity to dying epithelial cells may allow rapid access and uptake compared to recruited phagocytes. However, we may have underestimated the contribution of recruited macrophages or tumor-associated macrophages to the clearance of dying cells in the intestines. Targeting macrophages, or targeting both macrophages and epithelial cells may be more effective in altering overall efferocytosis rates in the intestinal tumor microenvironment. Future studies generating transgenic mice targeting other phagocytic cell types are required to address this question.

Our interest extended, beyond efferocytosis itself in CRC, to the accompanying inflammatory component. To boost efferocytosis in an anti-inflammatory manner, we used the *BELMO^Tg^* model. While we had previously seen remarkable improvements of intestinal inflammation in BELMO expressing mice using the DSS model of colitis (9), we did not observe either improved or worsened disease parameters in the AOM/DSS model of CRC. The main distinction between the studies is that the DSS model used by Morioka et al. was a model of acute inflammation induced by 3% DSS, while the current study focuses on chronic inflammation, and colitis-driven CRC. Here, mice were treated with multiple rounds of 1,5% DSS allowing for tumor development, and we examined cell death and cytokine levels at the end of the complete AOM/DSS treatment. At that stage, mice had recovered from the acute inflammatory phase induced by DSS and had developed tumors, displaying symptoms of chronic intestinal inflammation. It is possible that apoptotic cells are more abundant in the acute phase immediately following DSS treatment, when tissue damage is highest, and that BELMO confers a beneficial effect specifically under these conditions, rather than in the later chronic phase of inflammation. Similarly, inflammatory cytokine levels are likely higher during the acute phase, whereas in the chronic phase, the impact of BELMO may be diminished or absent.

To induce pro-inflammatory efferocytosis, we used the *Slc12a2^IEC-cKO^* model. Previously, Perry and colleagues found that deletion of *Slc12a2* enhances efferocytosis with a marked proinflammatory signature, both *in vitro* and *in vivo* (11). In this study, we knocked out *Slc12a2* in the intestinal epithelial cells. Consistent with the increased efferocytic capacity in *Slc12a2*-deficient cells seen previously (11), we observed that apoptotic cells were less abundant in the colons of *Slc12a2^IEC-cKO^* mice compared to wild-type mice. Despite the difference in apoptotic cell numbers, we could not detect increased pro-inflammatory cytokine levels produced in colon tissue from *Slc12a2^IEC-cKO^* mice. Similar to the *BELMO^IEC-Tg^* model, the timing of tissue sampling may not have been ideal to capture the effect of Slc12a2 deficiency on surrounding cytokine levels. Besides Slc12a2, Perry and colleagues introduced Slc12a4 as an obstructor of efferocytosis. We observed a similar pattern in our experiments, indicated by an increase in apoptotic cells in the colons of *Slc12a4^IEC-cKO^* mice.

Apart from its contribution to efferocytosis, there are a limited number of studies linking *Slc12a2* with CRC. Dan-Yang Chen and colleagues propose that *Slc12a2* is upregulated in CRC, however expression decreases with disease severity (20). Consistent with the idea that *Slc12a2* expression peaks at early stages of disease, Sugai et al. compared miRNA and mRNA expression between conventional adenomas, intramucosal cancer and invasive CRC (21). They only observed upregulation of *Slc12a2* in conventional adenomas, not in intramucosal or invasive CRC samples. By investigating miRNA/RNA networks in their samples, they suggest three miRNAs as potential regulators of *Slc12a2* expression. Expression of *hsa-miR-34a-5p*, *hsa-miR-15a-5p* and *hsa-miR-195-5p* are thought to be decreased in adenoma samples, resulting in increased expression of *Slc12a2*. In our experiments, we knocked out *Slc12a2* in intestinal epithelial cells and did not observe any differences in disease progression in its absence. As explained above for the *BELMO^IEC-Tg^* model, it is possible that intestinal epithelial cells are not the most relevant cell type to target, and that perhaps recruited macrophages or other immune cells could play a more significant role. Alternatively, as the SLC superfamily is quite large, with several closely related members, the function of Slc12a2 may be redundant and potentially compensated by related members, such as Slc12a1.

As for Slc12a4, the literature on its role in colorectal cancer is even more limited. Hankey et al. described an approximate 5-fold increase in *Slc12a4* expression in an APC-deficient murine CRC compared to healthy adjacent colon tissue (22). However, they did not observe the same increase in the AOM/DSS CRC model, while we do see an increase in *Slc12a4* expression in WT AOM/DSS treated mice vs WT untreated mice. Secondly, Papalazarou et al. found an indirect role for Slc12a4 in regulating serine uptake, alongside Slc6a14 as primary serine transporter, which is used by cancer cells to compensate for serine synthesis deficiency (23). Dual absence of *Slc12a4* and *Slc6a14* slowed down tumor growth and prolonged survival in a subcutaneous mouse model of CRC. Using individual knockouts of Slc12a4 in the AOM/DSS model, we did not observe altered CRC progression. This suggests that functional redundancy may take place *in vivo*. Accordingly, simultaneous targeting of Slc channels in an orthotopic CRC model could be interesting to pursue in future studies.

From this study, using three distinct approaches targeting efferocytic components in intestinal epithelial cells, we did not observe any alterations in disease progression or disease parameters in the context of the AOM/DSS model. Cancer is a very heterogenous disease both between patients and within one patient. Cancer cells can adapt themselves to new environments therefore, one tumor cell is not identical to another one. Although targeting efferocytosis as a stand-alone therapy does not alter disease progression, it might be that targeting efferocytosis is effective in combinatorial treatments. Future studies combining different types of cancer therapies or studies targeting efferocytic genes in other cell types within the colon or tumor may yield more knowledge on the role of efferocytosis in colorectal cancer development.

## Materials and methods

### Animal experiments

For AOM/DSS experiments in the BELMO^IEC-Tg^ model, *BELMO^fl-STOP-fl^ Villin1-Cre^Tg/+^* were used and BELMO*^fl-STOP-fl^* littermates were used as controls. For vehicle control conditions, we used *Villin1-Cre^Tg/+^* mice. In the *Slc12a2^IEC-cKO^* model, we used *Slc12a2^-/-^ Villin1-Cre^Tg/+^* mice as *Slc12a2^IEC-cKO^* and a mix of *Slc12a2^fl/fl^* and *Slc12a2^fl/+^* littermates as controls. For vehicle control conditions we used *Slc12a2^+/-^Villin1-Cre* mice. In the *Slc12a4^IEC-cKO^* model, we used *Slc12a^-/-^ Villin1-Cre^Tg/+^* mice as *Slc12a4^IEC-cKO^* and a mix of *Slc12a4^fl/+^* and *Slc12a4^fl/fl^* littermates as controls. Vehicle control mice were a mix of *Villin1-Cre^Tg/+^*, *Slc12a4^+/-^Villin1-Cre^Tg/+^* and *Slc12a4^-/-^Villin1-Cre^Tg/+^* mice. The *Slc12a2^fl/f^* and *Slc12a4^fl/fl^* mice were generated by the transgenic core facility at VIB, Ghent, Belgium, and *Villin-Cre* mice were obtained from Jax-Mice. AOM/DSS treatment was initiated at 8–10 weeks old, mice were weighed twice a week and were sacrificed 61 days after the start of the treatment. Mice were euthanized by CO_2_ inhalation.

*Rosa26-Zeb2^Tg^* mice were generated and described previously (24) and were kindly given to us by Prof. Lars Vereecke. To obtain simultaneous expression of Zeb2 and BELMO in the intestinal epithelium, *Rosa26-Zeb2^Tg/Tg^* were crossed to *BELMO^Tg^ Villin1-Cre* mice. *Zeb2^IEC-Tg^, BELMO/Zeb2^IEC-Tg^* and control mice were weighed 2 times per week from 4 weeks of age until the moment of sacrifice (max 25 weeks).

All mice had a C57BL/6 background. Mice were housed in individually ventilated cages at the VIB-UGent Center for Inflammation Research, in a specific pathogen-free (SPF) animal facility. Mice were housed under 14:10 (light:dark) light cycles, at 21°C and 60% humidity. These conditions are checked and maintained by vivarium staff daily. All experiments on mice were conducted according to institutional, national and European animal regulations. Animal protocols were approved by the ethics committee of Ghent University (EC file no. 2021-100, valid from 25/02/2022 and 2022-100, valid from 08/12/2022).

### AOM/DSS treatment

One single AOM (Santa Cruz biotechnology, SC-358746) injection of 10 mg/kg was given on day 0. One week after AOM injection, the first DSS (MP biomedical, 0216011080) cycle was initiated, followed by a 2-week rest period. The DSS cycle and rest period were repeated 2 more times after this resulting in a total of 3 DSS cycles. DSS was given in the drinking water at 1.5% m(g)/V(mL). Mice were weighed daily during DSS treatment and in the first four days after DSS treatment. During the remaining periods, mice were weighed twice a week.

### Stool score

The stool score was determined once per week. It consists of stool consistency measurements and detection of fecal blood. The presence of fecal blood was determined using the Hemocare Fecal Test (T3061). For bleeding, a score of 0 was assigned when no blood was detected, a score of 1 when small dots were visible on the Hemocare test, a score of 2 for larger positive parts on the hemocare test, a score of 3 when the whole stool was positive on the hemocare test and a score of 4 when blood could be seen be eye. For stool consistency, a score of 0 was assigned for hard, well-formed pellets, a score of 1 for pasty well-formed pellets, a score of 2 for pasty, semi-formed pellets, a score of 3 for watery semi-formed pellets and a score of 4 for liquid stools. The score for bleeding and consistency were added together and divided by two, resulting in the final stool score ranging from 0 for healthy mice to 4 for maximum pathology. To eliminate any diagnostic bias, the scoring investigator was blinded to the genotypes.

### Endoscopic analysis

Endoscopy was performed twice during the AOM/DSS procedure, once after the second and once after the third round of DSS treatment. High-resolution endoscopic videos were recorded with the Image 1S system (Karl Storz). Videos were scored using the MEICS scoring system (13). Mice were anesthetized with 4% isoflurane in oxygen for induction and were afterwards kept at 2.5% isoflurane in oxygen for maintenance of anesthesia during the procedure. In short, five parameters (thickness of the colon wall, vascular pattern, presence of fibrin, granularity of the surface and stool consistency) were given a score from 0 to 3 and then the average of all parameters is calculated and is used as the MEICS score. For thickness of the colon wall, a score of 0 was given when the arteries were easily visible and the lumen was smooth, a score of 1 when arteries were difficult to see, a score of 2 when arteries were not visible anymore and a score of 3 when the lumen is very narrow because of thickening of the colon wall. The vascular pattern was scored 0 when no bloodspots could be detected and arteries were easily visible, was scored 1 when only few, small bloodspots could be detected, was scored 2 when multiple blood spots could be detected and scored 3 when the lumen displayed gross bleeding. Presence of fibrin was given a score of 0 when no fibrin spots were present, 1 when only few fibrin spots were present, 2 when multiple fibrin spots were present and a score of 3 when large segments of white deposition were present in the lumen. The granularity of the surface was given a score of 0 when the lumen was completely smooth, 1 when the interior colon wall was uneven, 2 when protuberances were clearly visible, 3 when no smooth sections were left, protuberances were present everywhere. Finally, stool consistency was scored 0 for hard, well-formed pellets, 1 for semi-formed smoother pellets, 2 when pellets were breaking apart and stool was spread over de colon, and 3 was given for diarrhea.

Based on the endoscopic videos, tumors were scored on count and size. The size of the tumor was given a score from 1 to 5, based on the description of Becker et al (25). Videos where the colon wall was insufficiently visible (due to stool or blood) were excluded from the analysis.

### Histology

Mouse colons and ileums (most distal parts) were fixed overnight in 4% paraformaldehyde. Samples were dehydrated and embedded in paraffin as tubes. Cross sections of 5 μm were stained with hematoxylin and eosin staining using the Autostainer. Sections were digitally visualized using the Zeiss AxioScan Z1. For the TUNEL staining, we used the One-step TUNEL *in situ* apoptosis kit (Red, Elabscience E-CK-A322) according to the manufacturer’s protocol, with minor modifications. In short, tissues were deparaffinized and rehydrated and antigen retrieval was performed in target retrieval solution (Dako Agilent Target Retrieval Solution (Agilent, S169984-2) Slides were washed 3x with PBS and incubated with TdT equilibration buffer for 10 mins at 37°C. TdT equilibration buffer was removed and slides were incubated for 1 hour in the dark at 37°C with labeling working solution in a 1 in 25 dilution of the enzyme (2 μL TdT enzyme, 10 μL Label solution and 38 μL equilibration buffer). Samples were washed 3x with PBS and were stained with DAPI (made in-house) for 30 minutes. Samples were washed and mounted with PVA mounting media (made in-house). Slides were stored at 4°C in the dark until imaged.

### Cytokine production by colon explants

Distal colon was isolated. A 1 cm tissue piece was cut longitudinally and washed 3x with HBSS + 1% penicillin & streptomycin. After washing, tissue pieces were put in 500 μL RPMI 1640 + 1% penicillin & streptomycin and incubated for 18 hours at 37°C with 5% CO_2_. Supernatant was collected, centrifuged for 10 mins at 1300 x g at 4°C and frozen at -80°C until further use. Cytokine quantification in the supernatants was performed using a Bio-Plex multiplex immunoassay for simultaneous quantification of IL-6, IL-1α, IL-1β, and TNF, according to the manufacturer’s recommendations, with minor changes. Briefly, coupled beads were added to the plate. The plate was washed twice, standards, blanks and samples were added to the wells, and the plate was incubated on a shaker for 2 hours at RT. Plates were washed 3 times and incubated with detection antibodies for 1 hour at RT, while shaking. After incubation, the plate was washed 3 times and incubated with SA-PE for 30 mins at RT while shaking. The plate was washed and read by the Bio-Plex 200.

### RT-qPCR

Distal colon was preserved in RNAlater and kept at -20°C until further processing. 15–20 mg colon tissue was lysed in TRIzol using the TissueLyser. Further purification of total RNA was obtained by using the RNeasy Mini kit (Qiagen, 74104) according to the manufacturer’s instructions for total RNA purification from animal tissues. Total RNA concentration and quality was measured using the NanoDrop Spectrophotometer (Thermo Scientific) and converted to cDNA using the SensiFAST™ cDNA Synthesis kit (BIO-65054) according to the manufacturer’s instructions. Finally, gene expression was measured by the LightCycler^®^ 480 system using mouse-sequence specific TaqMan probes (Applied Biosciences) for the following genes: *B2m* (Mm00437762_m1), *Rpl13a* (Mm01612987_g1), *Cox4i* (Mm01250094_m1), *Matr3* (Mm01704913_g1), *BAI1* (Hs01105174_m1), *Elmo1* (Mm00519109_m1), *Slc12a2* (Mm00436546_m1), *Slc12a4* (Mm00486179_m1), *Il6* (Mm00446190_m1), *Il1a* (Mm00439620_m1), *Il1b* (Mm00434228_m1), *Tnf* (Mm00443258_m1), *Infg* (Mm01168134_m1), *Il10* (Mm01288386_m1), *Tgfb* (Mm01178820_m1).

### Efferocytosis measurements by IncuCyte

Macrophages were seeded one day prior to the assay in a 24-well plate. Apoptosis of Jurkat T cells was induced by UV-C irradiation (150mJ/cm2). Apoptotic targets were stained at 5x10^6^ cells/ml with pHrodo iFL Red at 2 μM in plain RPMI for 30 minutes at 37°C. After staining, the cell suspension was washed twice and resuspended in RPMI (10% FCS, 1%Pen-Strep) before adding to the phagocytes at a 5:1 target:phagocyte ratio. Plates were spun to bring the targets down onto the phagocytes (50 rcf, 1 min). Negative control wells were treated with Cytochalasin D at 1 μM 45 minutes to 1 hour prior to adding the targets. Phagocytosis was analyzed using the IncuCyte ZOOM of IncuCyte S3, by a change in pHrodo iFL Red intensity, outputting the Total fluorescent Integrated Intensity (GCU x μm2/Image).

## Data Availability

The raw data supporting the conclusions of this article will be made available by the authors, without undue reservation.
