# Supplementary material for: Modulating efferocytosis in the intestinal epithelial cells during colorectal cancer
Source: Front Oncol. 2026 Mar 9;16:1740918. doi: 10.3389/fonc.2026.1740918 (PMC13006223; doi:10.3389/fonc.2026.1740918)
Supplement: Supplementary file 1 [file DataSheet1.pdf]

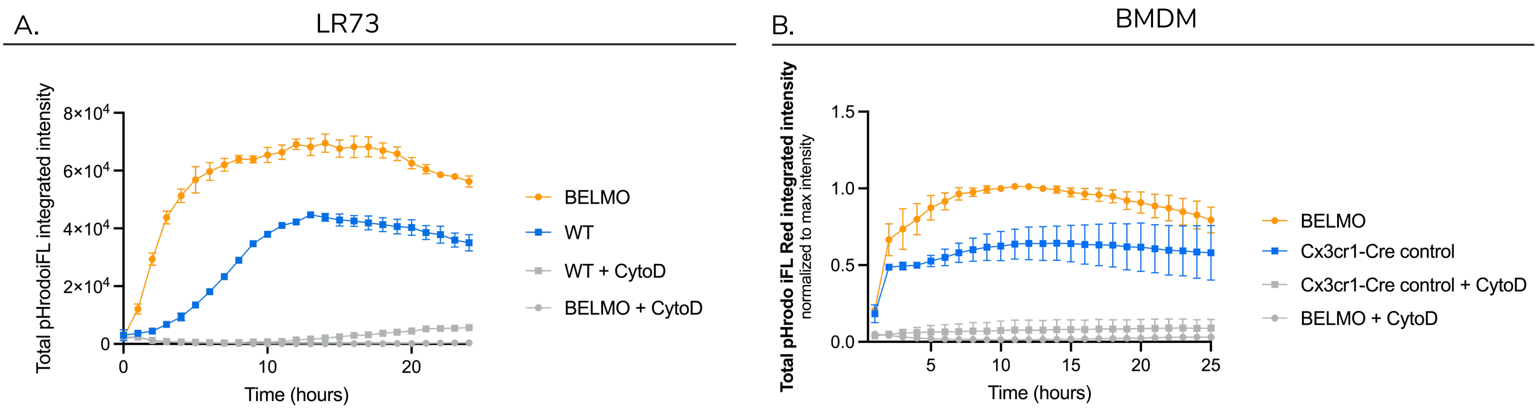

Figure S1: *In vitro* efferocytosis assay with BELMO^{Tg} cells. pHrodo Red-labeled apoptotic Jurkat cells were fed to (A) LR73 cells or (B) bone marrow-derived macrophages from Cx3cr1-Cre/BELMO^{Tg} mice in a 5 to 1 ratio. Fluorescence was acquired using IncuCyte live cell imaging up to 25 hours after co-culture. Cytochalasin D was used as a negative control. Data are represented as mean ± SEM. Each graph represents data from 3 or 4 independent replicates. CytoD = Cytochalasin D.

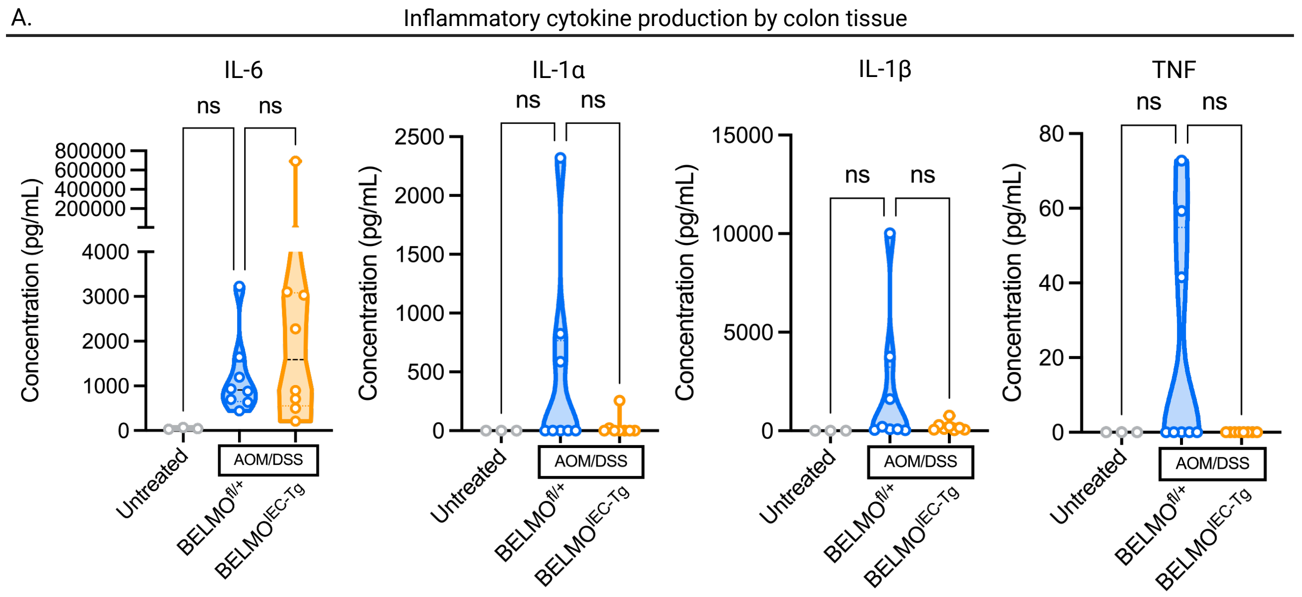

Figure S2: Cytokine production by colon tissue of BELMO^{IEC-Tg} mice (A) Colon tissue from BELMO^{IEC-Tg} mice treated with AOM/DSS and Cre-negative mice treated with AOM/DSS or untreated were isolated from the mice and put in culture overnight. Inflammatory cytokines were measured in the supernatant by ELISA. Ns = non-significant

A. BMDM

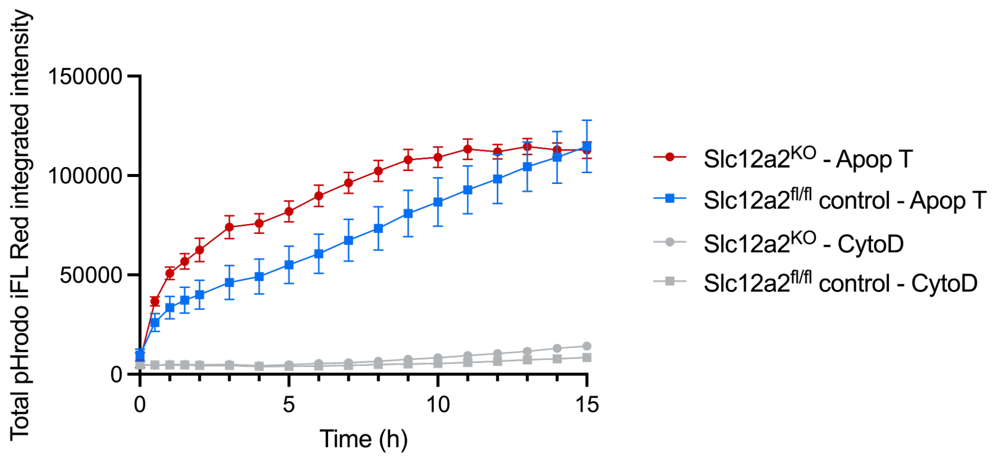

Figure S3: *Ex vivo* efferocytosis assay with *Slc12a2*^{KO} cells. pHrodo Red-labeled apoptotic Jurkat cells were fed to (A) LR73 cells or (B) bone marrow-derived macrophages from Cx3cr1-Cre/Slc12a2^{KO} mice in a 5 to 1 ratio. Fluorescence was acquired using IncuCyte live cell imaging up to 25 hours after co-culture. Cytochalasin D was used as a negative control. Data are represented as mean ± SEM. Each graph represents data from 3 or 4 independent replicates. CytoD = Cytochalasin D.

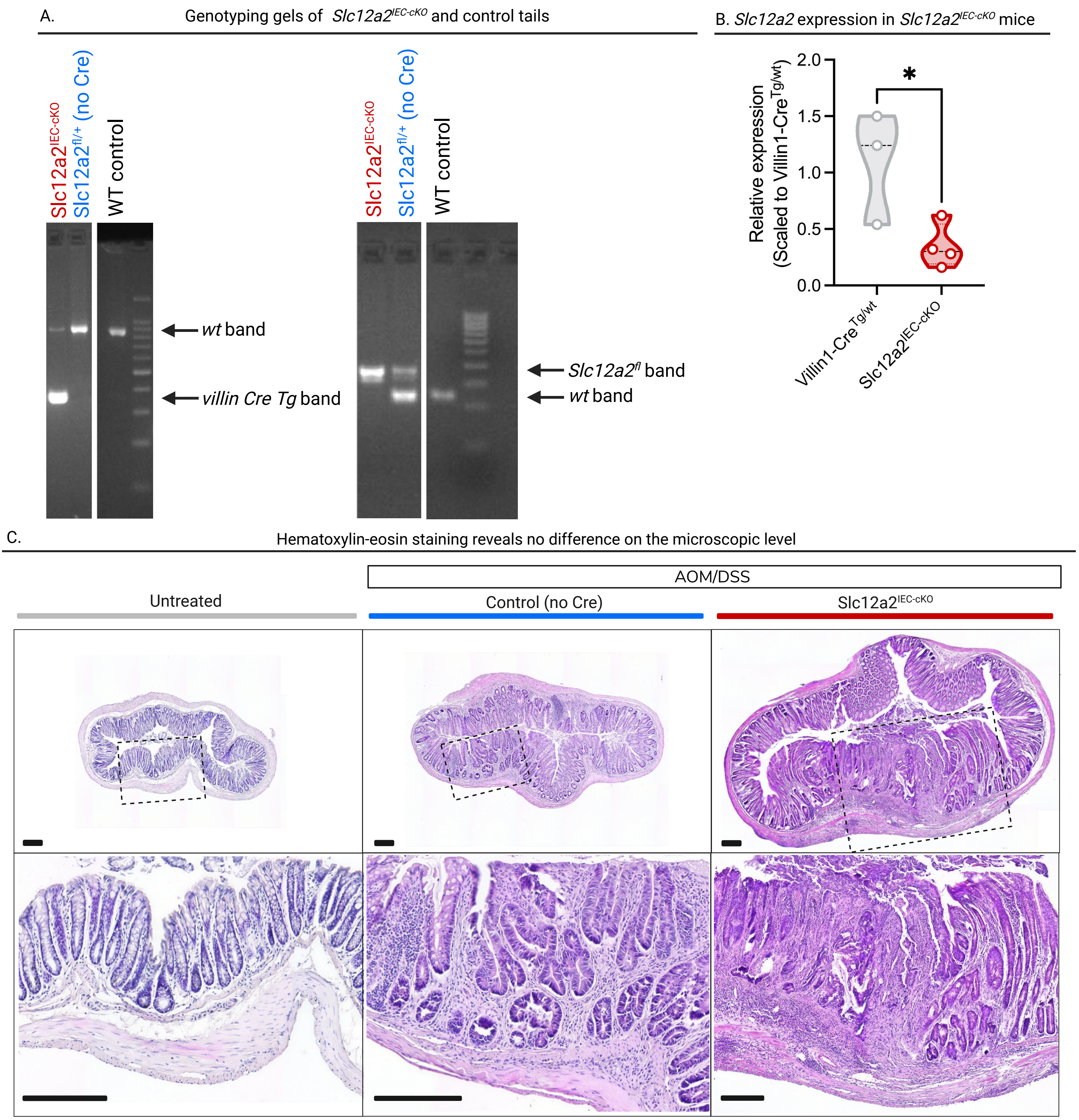

Figure S4: Characterization of Slc12a2$^{IEC\text{-}cKO}$ mice. (A) Gels showing genotyping of tail tissue from the Slc12a2$^{IEC\text{-}cKO}$ mice used for AOM/DSS experiments. (B) Validation of the Slc12a2 knock-out in the colon by RT-qPCR. (C) Representative Hematoxylin-eosin sections of Slc12a2$^{IEC\text{-}cKO}$ mice treated with AOM/DSS and Cre-negative mice with or without AOM/DSS treatment at the end of the treatment. Bottom: magnified images of the area defined by the dashed black boxes. Scale bar = 200 µm. * $p \leq 0.05$.

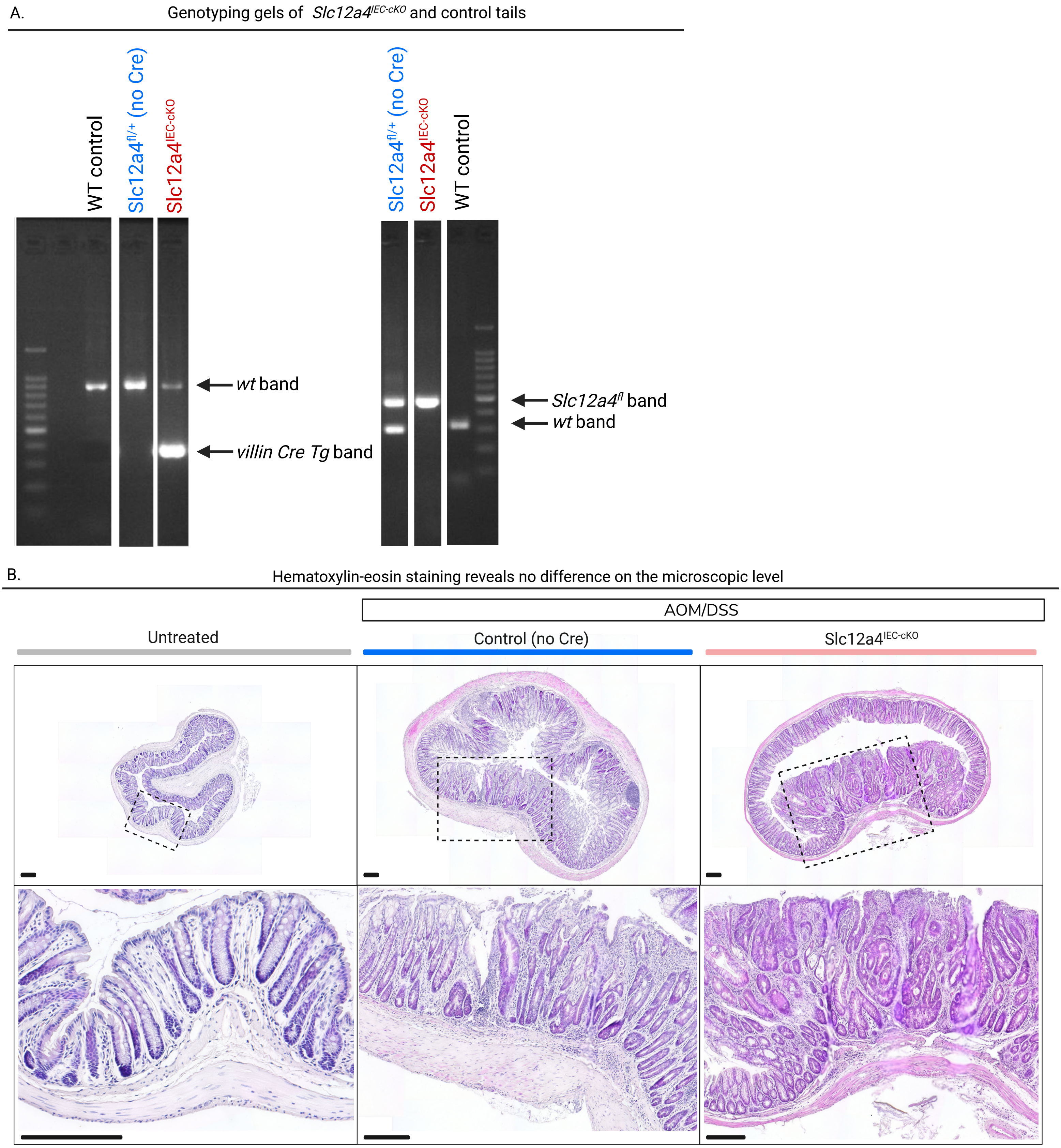

Figure S5: Characterization of Slc12a4$^{IEC\text{-}cKO}$ mice. (A) Gels showing genotyping of tail tissue from the Slc12a4$^{IEC\text{-}cKO}$ mice used for AOM/DSS experiments. (B) Representative images of Hematoxylin-eosin sections of Slc12a4$^{IEC\text{-}cKO}$ mice treated with AOM/DSS and Cre-negative mice with or without AOM/DSS treatment, at the end of the treatment. Bottom: magnified images of the area defined by the dashed black boxes. Scale bar = 200 μm.

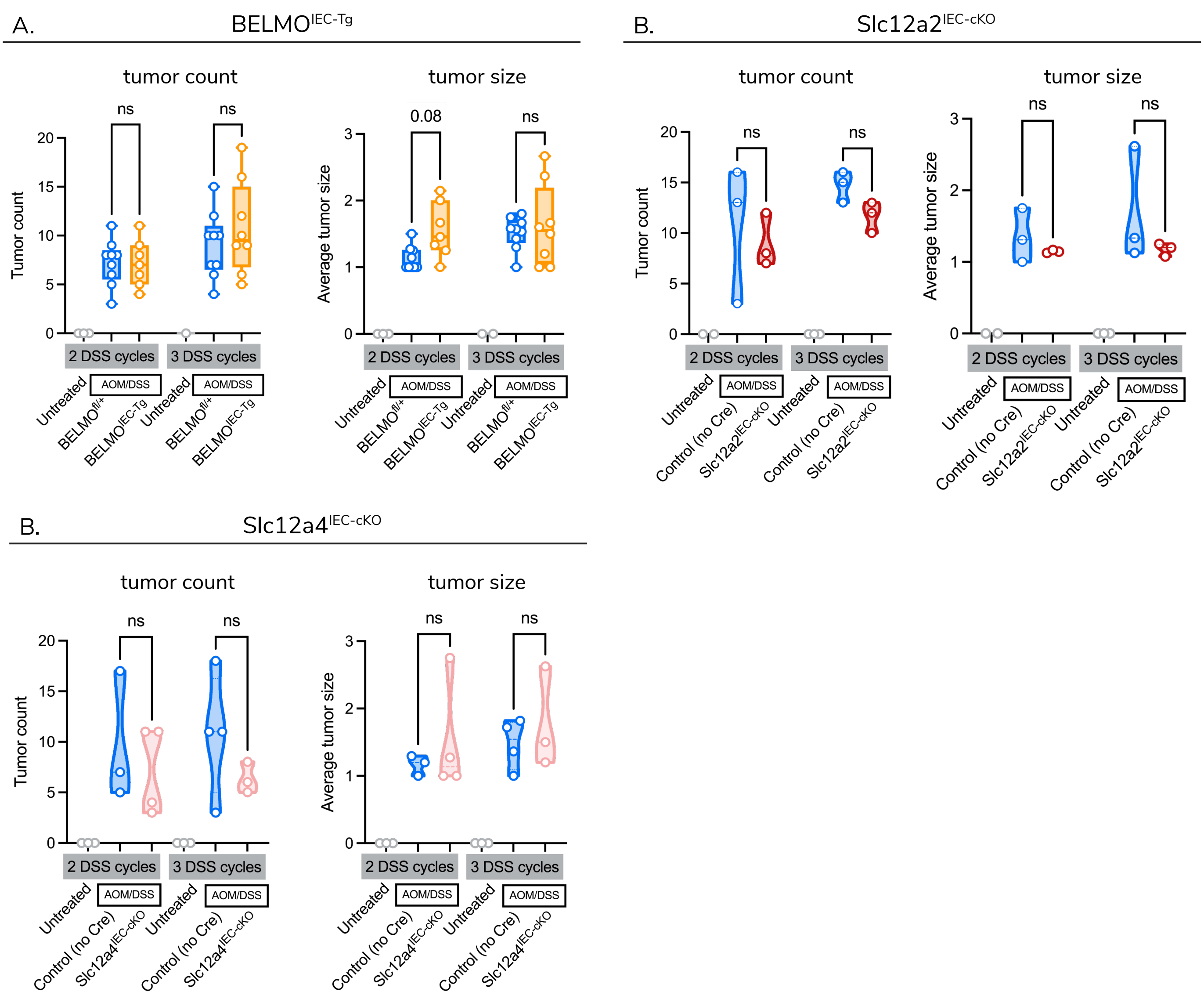

Figure S6: Tumor count and size in the AOM/DSS model of CRC. Colonoscopy was performed on untreated, and AOM/DSS-treated (A) BELMO$^{IEC\text{-}Tg}$ (n=8) and BELMO$^{fl/+}$ controls (n=9), (B) Slc12a2$^{IEC\text{-}cKO}$ (n=3) and Cre-negative mice (n=3) and (C) Slc12a4$^{IEC\text{-}cKO}$ (n=4) and Cre-negative mice (n=4) after recovery of the second (between day 40-42) and third (between day 56-58) DSS cycle. Tumor nodules were counted and scored on size. Each symbol represents one mouse. Data are represented as mean ± SEM. Statistical significance was determined by one-way ANOVA. ns = non-significant . Missing values are due to insufficient quality of the endoscopic video material.
